# Long-term trends in psychosocial working conditions in Europe—the role of labor market policies

**DOI:** 10.1093/eurpub/ckac038

**Published:** 2022-04-26

**Authors:** Mariann Rigó, Nico Dragano, Morten Wahrendorf, Johannes Siegrist, Thorsten Lunau

**Affiliations:** Institute of Medical Sociology, Centre for Health and Society, Medical Faculty, University of Düsseldorf, Düsseldorf, Germany; Institute of Medical Sociology, Centre for Health and Society, Medical Faculty, University of Düsseldorf, Düsseldorf, Germany; Institute of Medical Sociology, Centre for Health and Society, Medical Faculty, University of Düsseldorf, Düsseldorf, Germany; Senior Professorship on Work Stress Research, Medical Faculty, University of Düsseldorf, Düsseldorf, Germany; Institute of Medical Sociology, Centre for Health and Society, Medical Faculty, University of Düsseldorf, Düsseldorf, Germany; Institute for Social Research and Social Economy (iso), Saarbrücken, Germany

## Abstract

**Background:**

Employees have witnessed rising trend in work stress over the last few decades. However, we know a little about country differences in those trends. Our article fills this gap in the literature by examining heterogeneities in trends in working conditions by country groups defined by their amount of investment into labor market policy (LMP) programs. Additionally, we provide findings on differences in occupational inequalities between country groups.

**Methods:**

We use comparative longitudinal data of the European Working Conditions Surveys including cross-sectional information on employees from 15 countries surveyed in Waves 1995, 2000, 2005, 2010 and 2015. Estimation results are provided by three-way multilevel models with employees nested within country-years nested within countries. Our work stress measure is the proxy version of job strain based on the demand-control model.

**Results:**

Our regression results indicate that for employees in countries with the least LMP spending job strain increased by 10% from 1995 to 2015 compared to a smaller and insignificant change in middle- and high-LMP countries. In low-LMP countries, inequalities in job strain also widened during the studied period: the gap in job strain between the highest- and lowest-skilled increased by 60% from 1995 to 2015. This contrasts a stable gap in middle- and high-LMP countries.

**Conclusions:**

Our results direct the attention to the vulnerable position of the least skilled and highlight that LMP investments may buffer some of the adverse impacts of globalization and technological changes and effectively improve the labor market situation of the least skilled.

## Introduction

Long-term exposure to work-related stress can lead to a variety of adverse health- and labor market outcomes^1–4^ implying substantial losses for individuals, companies and the society.^5^ Work stress has been on a rise since the last few decades.^6–9^ An explanation for this general rising trend can be the profound structural changes of the labor markets. New technologies have appeared, the speed of globalization and digitalization has accelerated coupled with educational upgrading and demographic aging processes.^10^^,^^11^ These developments involved changes on the labor markets leading to new forms of work organizations characterized by higher degree of flexibility, job insecurity and complexity. Although these changes affected all European countries, their effects on experienced work stress may be different due to countries’ different labor market policies (LMPs), which aim to improve the functioning of the labor markets. Thus, they might have an influence on the trend of psychosocial working conditions. However, we are not aware of any studies testing this assumption.

A variety of mechanisms can explain the link between LMP investments and psychosocial working conditions at the country level.^12^^,^^13^ Passive labor market policies (PLMP) by ensuring an adequate level of living in case of job loss facilitate that (i) employees quit jobs with stressful working conditions and (ii) invest more time into job search in case of unemployment, thereby improving job match. Furthermore, PLMP investments into early retirement make it possible for older to work less or enter early retirement, which again reduces the average work stress level in a country. Investments into active labor market policies (ALMP) provide training opportunities for the unemployed, improving their skill set, which makes the match between their skills and the job requirements better. Therefore, we can assume that investments into LMP facilitate that individuals work in jobs with lower work-related stress.^12^ Positive associations between LMP spending and psychosocial working conditions were found both based on cross-sectional^14^ and longitudinal^12^ data. Furthermore, the general rising trend of work stressors in EU-15 countries was also documented in our previous paper.^6^ However, none of these studies investigated whether long-term trends in the perceived level of work stressors are different between countries based on their LMP investments and what the magnitudes of these differences are. Therefore, our first research question complements previous results by assessing heterogeneities in trends between countries grouped by their LMP spending.

Previous literature also provided evidence on differences in work stress by occupational position.^9^^,^^15^^,^^16^ Individuals in disadvantageous occupations were found to report higher level of work stress. Our previous paper also documented, in general, unfavorable long-term trends for low-skilled employees.^6^ Workers in different occupational positions could be differently impacted by global changes due to the varying effects of a number of processes, such as advancements in new technologies disfavoring routine occupations, educational upgrading, labor market deregulation or the increasing share of the service sector.^17–19^ Therefore, we delineate further our first research question by focusing on differences in work stress developments by occupational position within country groups. In case certain occupational groups benefit more from specific LMPs, these policies can also narrow or widen occupational inequalities in working conditions in countries where they are implemented.

First attempts have already been made to explore country differences in trends in work stress.^7^^,^^8^^,^^20^ However, the comparability of these studies is limited as they are based on short time period,^20^ not use validated measures of work stress^7^^,^^8^ or provide descriptive analysis based on raw data.^21^ Additionally, none of these studies link differences between countries to differences in labor market regimes.

## Methods

### Data

For the analysis comparative longitudinal survey data are needed, which include cross-sectional information on a large sample of employees surveyed from each country in several waves; thus, providing longitudinal information at the country level.^22^ Data from the European Working Conditions Survey (EWCS) incorporate these requirements. Our analysis is based on five waves of the EWCS from 1995, 2000, 2005, 2010 and 2015, and includes 15 countries participating in each wave (Belgium, Denmark, Germany, Greece, Spain, France, Ireland, Italy, Luxembourg, The Netherlands, Austria, Portugal, Finland, Sweden and UK). Its comparative longitudinal feature has important implications for our statistical analysis. First, we can study how psychosocial working conditions evolve over a 20-year period. Second, the hierarchical structure of the dataset makes it possible to carry out our analysis using multilevel modeling.^22^ The country sample sizes are around 1000 in each wave with a few exceptions where around 2000 employees are interviewed. Our analytical sample is restricted to those aged above 15 and below 65. Those outside this age interval may have particular work situations (e.g. work after pension age). For similar reasons, we also exclude persons working <8 h per week (1368) and those who were self-employed (12 935). The resulting database for our analysis includes 74 959 employee observations, sampled in 75 country-years in 15 countries.

### Variables, measurement

#### Working conditions

We used job strain based on the demand-control model^23^ to measure work stress. The model postulates that high levels of psychological demands coupled with low level of control lead to work-related stress termed as job strain. Associations between several health-related outcomes and job strain have been documented.^1^^,^^2^^,^^24^ Unfortunately, the full set of original questionnaire items is not included in the EWCS. Therefore, we constructed a shorter version and chose items as close as possible to the original ones similarly to previous papers.^6^^,^^20^ Job strain is the ratio of psychological demands and control. Control has two underlying constructs, skill discretion and decision authority. Responses to all single items have been standardized to have a range between 1 and 2, similarly to some previous studies.^6^^,^^25^ As such, all single items and the composite constructs of psychological demands, skill discretion and decision authority lie between 1 and 2, and job strain between 0.5 and 2. The list of underlying survey items and corresponding composite constructs are summarized by Supplementary table S1.

#### Occupation

We classified employees into four broad occupational groups based on the major groups of the ISCO-88 classification: Skill level 4—high-skilled clerical (HC, based on ISCO major groups 1–3); Skill level 3—low-skilled clerical (LC, based on ISCO major groups 4 and 5); Skill level 2—high-skilled manual (HM, based on ISCO major groups 6 and 7); and Skill level 1—low-skilled manual (LM, based on ISCO major groups 8 and 9). Armed forces (ISCO major group 0, *N* = 475) are excluded from the analysis as employees in this major group have heterogeneous skill levels making impossible to link them to one of the four broad skill levels.^26^

#### Investments into LMP programs

Similar to previous studies,^12^ we operationalize national LMPs by using spending into LMP defined as the sum of ALMP and PLMP expenditures as a percentage of GDP. LMP investments include e.g. job-search assistance, training, subsidized employment (active measures), unemployment insurance and disability benefits or investments into early retirement (passive measures).^27^ Our measure instead of focusing on its absolute value, captures the priority of LMP investments in the countries’ macroeconomic agenda. Previous studies^28^ often standardize expenditures in LMP by the country’s unemployment rate to adjust for countries’ need of such investments. Therefore, we group countries into three regimes by using the mean of this adjusted LMP in the examined period. Countries in the lowest tertile are called as low-LMP group, in the second tertile as middle-LMP group and in the highest tertile as the high-LMP group. The low-LMP group includes Greece, Spain, Italy, Portugal and the UK. Countries in the middle category are Germany, France, Ireland, Luxembourg and Finland. LMP spending is the highest in the group including Belgium, Denmark, the Netherlands, Austria and Sweden.

#### Other covariates

Following previous literature,^7^^,^^20^^,^^25^ to control for compositional differences between countries, we included control variables, such as age (dummies for three groups <30, 31–50 and >50), gender, contract type (indefinite, fixed-term, temporary and apprenticeship), sector (five groups by NACE categories) into our regressions. Additionally, we included GDP as a macro-level control variable.

### Statistical analyses

The EWCS has a three-level hierarchical structure where employees (Level 1) are nested within country-years (Level 2), which are nested within countries (Level 3). Using multilevel modeling, we regress the work stress indicators on a set of covariates. Our models incorporate a random intercept for countries and for country-years in addition to the individual error term. Multilevel estimation of nested data takes into account that observations within clusters are not independent. Therefore, it is superior to OLS, which assumes that each unit of observations is independent producing a bias in the standard error. We assess our first research question examining heterogeneity between country groups by adding an interaction term composed of the country group and wave dummies. To facilitate interpretation, we computed average predicted values of work stressors by country group. We tested between-group differences in the change over time by computing average marginal effects (AMEs) of a wave change in work stressors, and compared them between country groups. Additionally, we also computed the AME of changing the LMP regime within each wave. To address our second research question delineating trends by occupational groups within countries, we added three-way interactions of wave, occupational and country-group dummies. Similar to the previous case, predicted values by occupation in each country group, and AMEs were computed. We examined heterogeneity in the change over time within each broad occupational group by country regime, and assessed differences between occupational groups over time in each regime.

## Results

### Descriptive statistics

Table 1 includes our main macroeconomic variable of interest, the investments into LMP programs as percent of GDP for each country. Its value adjusted by the unemployment rate is used to capture the priority of LMP spending in the country’s macroeconomic agenda and serves the basis of classifying countries. The raw descriptive statistics by country group are included in the Supplementary table S2.

**Table 1:** Macroeconomic variables by country

		1995		2000		2005		2010		2015	
		LMP (% of GDP)	Unemp rate	LMP (% of GDP)	Unemp rate	LMP (% of GDP)	Unemp rate	LMP (% of GDP)	Unemp rate	LMP (% of GDP)	Unemp rate
High-LMP	Belgium	3.40	9.7	2.72	6.9	2.75	8.5	2.73	8.3	2.23	8.5
	Denmark	5.81	6.7	3.88	4.3	3.50	4.8	3.36	7.5	2.92	6.2
	The Netherlands	3.43	8.3	2.40	3.7	2.53	5.9	2.16	5.0	2.30	6.9
	Austria	1.83	4.2	1.51	3.9	1.89	5.6	2.00	4.8	2.04	5.7
	Sweden	3.78	8.8	2.68	5.6	2.11	7.7	1.60	8.6	1.56	7.4
Middle-LMP	Germany	3.21	8.2	2.83	7.9	2.73	11.2	1.80	7.0	1.15	4.6
	France	2.47	10.2	2.71	8.6	2.67	8.9	2.69	9.3	2.70	10.4
	Ireland	3.54	12.3	1.43	4.5	1.29	4.6	3.51	14.6	1.70	10.0
	Luxembourg	0.74	2.9	0.58	2.2	1.11	4.6	1.28	4.6	1.29	6.5
	Finland	4.94	15.4	2.74	9.8	2.52	8.4	2.54	8.4	2.78	9.4
Low-LMP	Greece	0.62	n.a.	0.61	11.2	0.46	10	0.93	12.7	0.69	24.9
	Spain	2.70	20.7	2.02	11.9	2.06	9.2	3.80	19.9	2.43	22.1
	Italy	1.18	11.2	1.16	10	1.13	7.7	1.64	8.4	1.70	11.9
	Portugal	1.18	7.9	1.26	5.1	1.73	8.8	1.98	12	1.84	12.6
	UK	0.87	8.5	n.a.	5.4	0.21	4.8	0.35	7.8	n.a.	5.3

Notes: LMP: expenditure on labor market policies (ALMP+PLMP, Categories 2–9) in percentage of GDP.

Source: OECD public expenditure and participant stocks on LMP dataset (https://stats.oecd.org/). Unemployment rate: unemployment in percent of active population.

Source: Eurostat une_rt_a dataset (https://appsso.eurostat.ec.europa.eu/nui/show.do?dataset=une_rt_a&lang=en).

### Analysis by LMP spending

Regression results showing predicted values and AMEs are depicted by table 2.

**Table 2:** Predicted values of w ork stressors and AMEs by country group based on linear multilevel models

	1995	2000	2005	2010	2015	AME 2005 vs. 1995 (*P*-value)	AME 2015 vs. 2005 (*P*-value)
**Job strain (*N* = 74 959)**							
Low-LMP	0.878 (0.834–0.922)	0.922 (0.879–0.965)	0.937 (0.898–0.977)	0.929 (0.890–0.968)	0.972 (0.932–1.011)	0.059 (0.001)	0.035 (0.029)
Middle-LMP	0.869 (0.830 − 0.908)	0.911 (0.872 − 0.950)	0.907 (0.866 − 0.948)	0.942 (0.897 − 0.986)	0.895 (0.850 − 0.939)	0.038 (0.035)	−0.012 (0.456)
High-LMP	0.864 (0.825 − 0.903)	0.883 (0.843 − 0.922)	0.905 (0.865 − 0.944)	0.898 (0.856 − 0.940)	0.899 (0.859 − 0.940)	0.041 (0.016)	−0.006 (0.724)
AME high vs. low (*P*-value)	−0.014 (0.634)	−0.039 (0.168)	−0.033 (0.262)	−0.031 (0.299)	−0.073 (0.014)		
**Psychological demand (*N* = 74 959)**							
Low-LMP	1.372 (1.314 − 1.430)	1.388 (1.330 − 1.445)	1.439 (1.387 − 1.492)	1.415 (1.363 − 1.466)	1.474 (1.421 − 1.526)	0.067 (0.004)	0.035 (0.111)
Middle-LMP	1.395 (1.343 − 1.447)	1.427 (1.375 − 1.479)	1.440 (1.386 − 1.494)	1.462 (1.403 − 1.521)	1.431 (1.372 − 1.490)	0.045 (0.070)	−0.009 (0.684)
High-LMP	1.431 (1.380 − 1.483)	1.445 (1.393 − 1.498)	1.487 (1.435 − 1.540)	1.452 (1.396 − 1.507)	1.469 (1.415 − 1.523)	0.056 (0.016)	−0.018 (0.401)
AME high vs. low (*P*-value)	0.059 (0.123)	0.058 (0.124)	0.048 (0.208)	0.037 (0.347)	−0.005 (0.902)		
**Skill discretion (*N* = 74 959)**							
Low-LMP	1.571 (1.533 − 1.609)	1.532 (1.495 − 1.569)	1.577 (1.543 − 1.611)	1.549 (1.515 − 1.582)	1.559 (1.525 − 1.593)	0.006 (0.704)	−0.018 (0.214)
Middle-LMP	1.648 (1.614 − 1.681)	1.604 (1.571 − 1.638)	1.631 (1.596 − 1.667)	1.590 (1.552 − 1.629)	1.634 (1.595 − 1.672)	−0.016 (0.329)	0.002 (0.884)
High-LMP	1.669 (1.636 − 1.703)	1.662 (1.628 − 1.696)	1.685 (1.651 − 1.719)	1.660 (1.624 − 1.696)	1.675 (1.640 − 1.710)	0.016 (0.317)	−0.010 (0.510)
AME high vs. low (*P*-value)	0.098 (0.000)	0.130 (0.000)	0.108 (0.000)	0.111 (0.000)	0.116 (0.000)		
**Decision authority (*N* = 74 959)**							
Low-LMP	1.664 (1.606 − 1.723)	1.603 (1.545 − 1.660)	1.610 (1.557 − 1.664)	1.616 (1.563 − 1.669)	1.598 (1.544 − 1.651)	−0.054 (0.012)	−0.013 (0.521)
Middle-LMP	1.680 (1.627 − 1.733)	1.654 (1.601 − 1.707)	1.660 (1.604 − 1.715)	1.635 (1.575 − 1.694)	1.674 (1.615 − 1.734)	−0.021 (0.367)	0.015 (0.476)
High-LMP	1.743 (1.690 − 1.796)	1.729 (1.676 − 1.783)	1.712 (1.659 − 1.765)	1.688 (1.632 − 1.744)	1.702 (1.648 − 1.757)	−0.031 (0.140)	−0.010 (0.615)
AME high vs. low (*P*-value)	0.079 (0.044)	0.127 (0.001)	0.102 (0.009)	0.072 (0.074)	0.105 (0.009)		

Notes: Predicted values based on multilevel model regressions with three levels (Level 1: individual, Level 2: country-years and Level 3: country). Covariates included in the regression: gender, age (<30, 30–50 and 50<), occupation (HC, LC, HM and LM), contract type (indefinite, fixed term, temporary, apprenticeship and other), NACE (‘agriculture, hunting, forestry and fishing’, ‘industry’, ‘services’, ‘public administration and defence; compulsory social sec’ and ‘other services’), GDP (in current US-Dollar, https://data.worldbank.org/indicator/NY.GDP.MKTP.CD), two-way interactions of wave dummies and country groups (low-LMP, middle-LMP and high-LMP). The 95% confidence intervals in parenthesis. AME: *P*-values in parenthesis. Sample: EU15, waves included: 1995, 2000, 2005, 2010 and 2015.

A clear gradient is visible from high- to low-LMP countries with increasing job strain, and an opposite pattern in case of skill discretion and decision authority. In case of psychological demands, differences between countries are mostly insignificant. Turning to the examination of trends, job strain has increased the most in low-LMP countries during the studied period. It increased from its value of 0.878 in 1995 by 10% until 2015. Job strain has also increased between 1995 and 2005 in middle- and high-LMP countries; however, a subsequent increase took place only in countries with low priority of LMP investments. As indicated by the last row of each section in table 2 (AME high vs. low), the gap in job strain between low- and high-LMP countries widened over time: its value became almost five times larger in magnitude and statistically significant in 2015 compared to 1995. The intensification of job strain from 1995 to 2005 is mostly driven by increases in psychological demands. Additionally, in case of low-LMP countries a deterioration of decision authority is observed from 1995 to 2005.

### Analysis by occupational position and LMP spending

**Figure 1:**
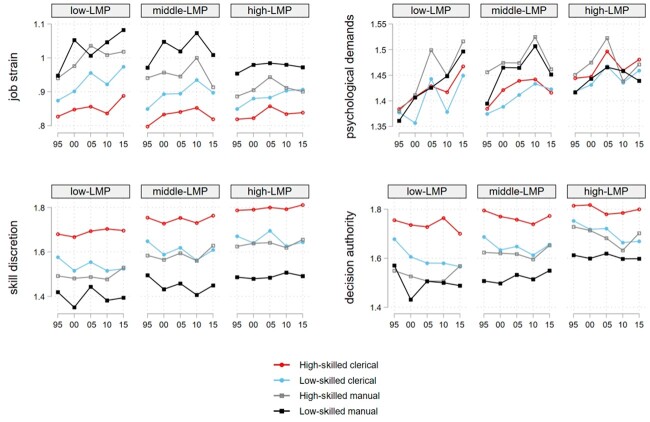
Predicted values of work stressors by occupation and LMP spending

Figure 1 provides a visual illustration of the average predicted values of work stressors by occupational group in the different LMP regimes. The estimates and AMEs for job strain are shown in table 3, while for the other work stressors they are provided in the Supplementary tables S3–S5. We have computed the AMEs of a wave change from 1995 to 2005 (and from 2005 to 2015) depicted in the last two columns of the tables and tested whether they were significantly different between occupational or country groups. The last row of each subsection in the tables includes the AME of changing the skill level but holding wave and country-group constant (AME LM vs. HC).

**Table 3:** Predicted values of job strain by occupation and country group

	1995	2000	2005	2010	2015	AME 2005 vs. 1995 (*P*-value)	AME 2015 vs. 2005 (*P*-value)
**Job strain**							
**Low-LMP**							
High-skilled clerical	0.827 (0.781–0.873)	0.848 (0.802 − 0.893)	0.856 (0.814 − 0.898)	0.836 (0.795 − 0.877)	0.888 (0.847 − 0.929)	0.029 (0.144)	0.032 (0.078)
Low-skilled clerical	0.874 (0.828 − 0.920)	0.901 (0.857 − 0.946)	0.956 (0.913 − 0.998)	0.922 (0.881 − 0.963)	0.974 (0.933 − 1.015)	0.081 (0.000)	0.018 (0.313)
High-skilled manual	0.940 (0.893 − 0.987)	0.976 (0.930 − 1.022)	1.036 (0.990 − 1.082)	1.009 (0.965 − 1.053)	1.018 (0.975 − 1.062)	0.096 (0.000)	−0.017 (0.425)
Low-skilled manual	0.948 (0.901 − 0.994)	1.053 (1.007 − 1.098)	1.007 (0.965 − 1.049)	1.046 (1.004 − 1.089)	1.083 (1.041 − 1.124)	0.059 (0.004)	0.076 (0.000)
AME LM vs. HC (*P*-value)	0.121 (0.000)	0.205 (0.000)	0.151 (0.000)	0.210 (0.000)	0.194 (0.000)		
**Middle-LMP**							
High-skilled clerical	0.798 (0.757 − 0.839)	0.833 (0.792 − 0.874)	0.841 (0.798 − 0.883)	0.853 (0.807 − 0.898)	0.819 (0.774 − 0.865)	0.043 (0.033)	−0.021 (0.235)
Low-skilled clerical	0.849 (0.809 − 0.890)	0.893 (0.852 − 0.934)	0.895 (0.852 − 0.938)	0.935 (0.890 − 0.980)	0.897 (0.851 − 0.943)	0.045 (0.024)	0.003 (0.892)
High-skilled manual	0.941 (0.899 − 0.983)	0.957 (0.915 − 0.999)	0.945 (0.899 − 0.991)	1.000 (0.952 − 1.048)	0.914 (0.865 − 0.963)	0.004 (0.846)	−0.032 (0.151)
Low-skilled manual	0.971 (0.929 − 1.014)	1.048 (1.006 − 1.090)	1.019 (0.975 − 1.064)	1.074 (1.027 − 1.120)	1.009 (0.962 − 1.056)	0.048 (0.029)	−0.011 (0.603)
AME LM vs. HC (*P*-value)	0.174 (0.000)	0.215 (0.000)	0.179 (0.000)	0.221 (0.000)	0.190 (0.000)		
**High-LMP**							
High-skilled clerical	0.819 (0.778 − 0.860)	0.822 (0.782 − 0.863)	0.857 (0.817 − 0.898)	0.834 (0.792 − 0.877)	0.839 (0.797 − 0.880)	0.038 (0.038)	−0.019 (0.275)
Low-skilled clerical	0.849 (0.808 − 0.890)	0.880 (0.840 − 0.921)	0.883 (0.842 − 0.924)	0.903 (0.860 − 0.946)	0.907 (0.865 − 0.949)	0.034 (0.075)	0.024 (0.187)
High-skilled manual	0.886 (0.843 − 0.930)	0.905 (0.862 − 0.948)	0.943 (0.898 − 0.989)	0.912 (0.865 − 0.958)	0.901 (0.854 − 0.947)	0.057 (0.013)	−0.043 (0.064)
Low-skilled manual	0.954 (0.911 − 0.996)	0.980 (0.938 − 1.022)	0.985 (0.940 − 1.029)	0.980 (0.936 − 1.024)	0.972 (0.929 − 1.016)	0.031 (0.156)	−0.012 (0.556)
AME LM vs. HC (*P*-value)	0.135 (0.000)	0.157 (0.000)	0.127 (0.000)	0.146 (0.000)	0.134 (0.000)		
Observations	74 959						

Notes: Predicted values based on multilevel model regressions with three levels (Level 1: individual, Level 2: country-years and Level 3: country). Covariates included in the regression: gender, age (<30, 30–50 and 50<), contract type (indefinite, fixed term, temporary, apprenticeship and other), NACE (‘agriculture, hunting, forestry and fishing’, ‘industry’, ‘services’, ‘public administration and defence; compulsory social sec’ and ‘other services’), GDP (in current US-Dollar, https://data.worldbank.org/indicator/NY.GDP.MKTP.CD), three-way interactions of wave dummies, isco categories (HC, LC, HM and LM) and country groups (low-LMP, middle-LMP and high-LMP). The 95% confidence intervals in parenthesis. AME: *P*-values in parenthesis. Sample: EU15, waves included: 1995, 2000, 2005, 2010 and 2015.

The predicted values in job strain indicate that employees in the lowest occupational position (LM) in low-LMP countries experienced the largest deterioration of psychosocial working conditions. They are the only group with significant increase in job strain both from 1995 to 2005 and from 2005 to 2015. For them, job strain increased from its value of 0.948 in 1995 by 0.135 units corresponding to more than a 14% increase by 2015. Furthermore, in low-LMP countries, the low-skill/high-skill gap in the predicted value of job strain increased significantly from its initial value of 0.121 by more than 60% (AME LM vs. HC). Though a social gradient with respect to skill level is also detected in the other country groups, the gap in those did not widen significantly over time. The deterioration in job strain among the least skilled in low-LMP countries from 2005 to 2015 is driven by a large increase in psychological demands (Supplementary table S3) and a parallel decrease in skill discretion (Supplementary table S4). While psychological demands increased for all groups from 1995 to 2005, a further increase took place only in low-LMP countries among the least skilled. Simultaneously, while we could not detect significant changes in skill discretion over time, least skilled in low-LMP countries stand out experiencing a significant decrease in skill discretion from 2005 to 2015.

## Discussion

Our study analyzed heterogeneities in long-term trends in work stress between 1995 and 2015. Our main findings indicate that low-skilled employees experienced the largest deterioration of job strain between 1995 and 2015 in low-LMP countries. Furthermore, inequalities between the highest- and lowest-skilled also expanded in those countries. On the other hand, such an aggravation of inequalities was not detected in middle- and high-LMP countries.

Our results are congruent with findings from previous papers and complement those. Previous studies suggested a deterioration of working conditions from the 1990’s^6^^,^^7^^,^^21^ and also indicated country differences.^7^^,^^20^^,^^21^ Some studies^7^^,^^21^ found worsening trend in working conditions in Mediterranean countries. Lopes et al.^21^ and the flagship report of Eurofound^29^ both pointed out that Northern countries are characterized by high levels of autonomy and high levels of work intensity with work intensity having a peak in 2005. Our results point to the same direction and also relate those changes to differences in LMP investments.

Turning to the results by occupational and country group, previous literature provided evidence that the deterioration of working conditions was larger among the least skilled.^6^^,^^20^^,^^21^ Our results add to these findings by pointing out that the least skilled fared significantly worse in low-LMP countries. Specifically, while they experienced more than a 14% increase in job strain from 1995 to 2015 in low-LMP countries, the same occupational group in middle- and high-LMP countries saw a small and insignificant movement in job strain. Regarding occupational differences in work stress by country groups, a previous study^21^ provided indication that a polarization trend between the high- and low-skilled in job quality took place, with the exception of Scandinavian countries where the quality of jobs were found to be more egalitarian. Our results point to the same direction indicating widening gap in job strain between the least and the highest skilled only in low-LMP countries from 1995 to 2015.

Furthermore, our results indicate a parallel increase in psychological demands in low-LMP countries among the low-skilled group. This group includes workers performing simple and routine tasks often requiring physical effort (major group 9) and workers operating and monitoring highly automated, industrial machinery (major group 8). Globalization and technological change are most likely to impact them adversely limiting their employment and job potentials.^11^ Thus, maintaining their skills, upskilling and reskilling are essential tools for them to ‘navigate through an ever-changing, technology-rich work environment’.^11^ Our findings indicate that LMP investments, through several possible mechanisms, had a beneficial long-term impact on the working conditions of the low skilled.

Our study has several limitations. First, our choice of work stress indicators was constrained by the available survey items in EWCS. It was only possible for work stress constructs of the demand-control model to construct comparable items throughout five consecutive waves. We also examined the work stress indicator based on the effort-reward imbalance model^30^ where items were only available for the last three waves. These results point qualitatively to the same direction. As our aim was to assess long-term trends, we decided not to include those results. Even for job strain, we could only construct a proxy version. However, previous literature provided evidence on the close correspondence between the original constructs and their proxy or partial versions.^31^^,^^32^ Second, response rates are country-specific, they may change over time influencing our results. This selective non-response might be a problem if there are systematic differences between respondents and non-respondents in terms of unobservable characteristics related to work stress. We carried out further analysis (correlation between response rates and work stressors, regressions adjusted for the response rate), which did not indicate that it would bias our results. Third, we classified countries into groups based on their average LMP investments from 1995 to 2015. During this relatively long period, countries could have experienced marked changes in their LMPs, which might have led to marked changes in experienced level of work stress. Pooling such countries together with countries having relatively stable LMPs may distort the results. To check whether such marked changes took place, we repeated the grouping based on the last 10 years. The groups stayed the same except for the position of Finland and Sweden (with no significant impact on the results). Additionally, it needs to be emphasized that our stratified analysis by country group is not indicative of any causal relationship between work stressors and LMP spending. However, it was not our aim. Instead, our focus was to examine whether countries with higher priorities in LMP spending were more successful to maintain better working conditions during the last 20 years and whether the gap between low- and high-LMP countries remained stable or widened over time. A widening gap might suggest that countries with higher priority of LMP investments in their macroeconomic agenda did a better job to counteract the negative consequences of globalization and technological trends on working conditions. However, heterogeneities between country groups may be also due to other country specificities not captured in our analysis, e.g. differences in the strengths of trade unions or differences in exposure to global labor market changes.

Despite these limitations, this article has several contributions. Our study is the first quantifying long-term changes in validated measures of work stressors by an important country-level policy tool previously linked to the perceived level of work stressors both using cross-sectional^14^ and longitudinal^12^ data. Our analysis utilizing the exceptional data structure of EWCS with a longitudinal and a cross-sectional character uses an advanced statistical method to assess the evolution of work stress. Previous attempts providing evidence on trends in work stressors by countries were based on descriptive statistics.^7^^,^^21^ Additionally, we carried out our statistical analysis relying on a proxy of job strain, which has been previously linked to detrimental health outcomes. Previous studies using similar operationalization of work stress focused only on two waves^20^ or used more waves but relied on a different conceptualization of work stress.^7^^,^^21^ Furthermore, our analysis advances on previous papers by focusing on differences in work stress by distinct national labor policies. Previous works often grouped countries into welfare regimes including bundles of macro policy tools^21^ or analyzed countries separately.^20^

Additionally, our analysis moves one step further and scrutinizes trends by occupation within each country group. This facilitates identifying social groups being the most vulnerable to the changes in labor markets over the past decades and helps our understanding of the mechanisms behind. Our recent study^6^ provided evidence that in general least skilled employees witnessed the least favorable trends in work stressors over the past decades characterized by high levels of psychological demand, low levels of control resulting in an elevated amount of work stress. This study complements our previous findings showing that their relative position was less detrimental in countries having high priority of LMP investments. While occupational differences in terms of job strain between the highest- and lowest-skilled widened in low-LMP countries, they have not changed throughout the whole period in middle- and high-LMP countries. Therefore, our results might give first indication that investment into LMP programs might have counteracted some of the adverse consequences of global labor market changes.

## Policy conclusions

Work stress has established linkages to a variety of health outcomes. Therefore, our results on trends in work stressors have important policy relevance. Our findings direct the attention to the vulnerable position of the least skilled and also to the use of macro-level policy instruments targeting this occupational group. Among the potential available instruments, allocating a higher share of national-level spending into LMP might be an efficient one to improve the situation of the least skilled.

## Supplementary data

Supplementary data are available at *EURPUB* online.

## Funding

This project was funded by the Deutsche Forschungsgemeinschaft (DFG, German Research Foundation—grant number: 392132829 ‘LU 2211/1-1’).

## Disclaimer

The funders of this study were neither involved in its design nor in collection, analysis, interpretation of data or in writing of the manuscript.

*Conflicts of interest*: None declared.

## Data availability statement

The EWCS datasets are stored with the UK Data Service (UKDS) in Essex, UK and are publicly available via their website (https://ukdataservice.ac.uk/). Users are required to be registered with the UK Data Service. Users who register have to accept the End User Licence (EUL), which is agreed to during the registration process.

## Ethics approval and consent to participate

The statistical analysis of the EWCS data was confirmed by the Ethics Committee of the Medical Faculty of the Heinrich-Heine-University Düsseldorf (2018-40-RetroDEuA).

Key points

- This study examines, for the first time, long-term trends in EU 15 countries in psychosocial working conditions differentiated by skill level and labor market policy (LMP) investments.
- Least skilled in countries with the lowest priority of LMP investment experienced the most deteriorating trend in job strain.
- Inequalities in working conditions between the lowest and highest skilled widened over time in countries with low priority of LMP investments.
- Widening inequalities between the low- and high-skilled are not detected in countries with middle and high priority of LMP spending.
- Our findings highlight the vulnerable position of the least skilled and suggest that LMP investments may buffer some of the adverse impacts of globalization and technological changes.

## Supplementary Material

ckac038_Supplementary_Data
