# Supplementary material for: Long-term trends in psychosocial working conditions in Europe—the role of labor market policies
Source: Eur J Public Health. 2022 Apr 26;32(3):384–91. doi: 10.1093/eurpub/ckac038 (PMC9159322; doi:10.1093/eurpub/ckac038)
Supplement: ckac038_Supplementary_Data [file ckac038_supplementary_data.docx]

**Online Supplementary Material**

**Table A1. List of underlying EWCS survey items.**

| **Work stress measure** | **EWCS item** | **Response categories** |
| --- | --- | --- |
| **Job strain (Psychological demands/Control)** | |  |
| **Psychological demand** | Does your work including working at a very high speed? | 1 (never) to 7 (all of the time) |
|  | Does your work including working to tight deadlines? | 1 (never) to 7 (all of the time) |
| **Skill discretion** | Does your job include learning new things? | 1 (no) to 2 (yes) |
|  | Does your job include solving complex tasks? | 1 (no) to 2 (yes) |
|  | Does your job include monotonous tasks | 1 (no) to 2 (yes) |
|  | Does your job include repetitive tasks of less than 10 minutes? | 1 (no) to 2 (yes) |
| **Decision authority** | Are you able to choose the order of the tasks? | 1 (no) to 2 (yes) |
|  | Are you able to choose the methods of your work? | 1 (no) to 2 (yes) |
|  | Are you able to choose the rate or speed of your work? | 1 (no) to 2 (yes) |

Similarly to our previous paper (1) and following Niedhammer et al. (2), survey items were standardized in the following way:

$${item\_st}_{i}=1+\frac{\left( {item\_orig}_{i}-1 \right)}{x-1}$$

where *x* refers to the number of original response categories.

**Table A2: Descriptive statistics of the analytical sample**

|  |  |  | **1995** | **2000** | **2005** | **2010** | **2015** |
| --- | --- | --- | --- | --- | --- | --- | --- |
| **Job strain** | **low-LMP** | mean (w) | 0.89 | 0.92 | 0.95 | 0.92 | 0.95 |
|  |  | *sd (w)* | *0.28* | *0.29* | *0.30* | *0.28* | *0.29* |
|  | **middle-LMP** | mean (w) | 0.89 | 0.91 | 0.93 | 0.95 | 0.89 |
|  |  | *sd (w)* | *0.30* | *0.30* | *0.30* | *0.30* | *0.27* |
|  | **high-LMP** | mean (w) | 0.87 | 0.88 | 0.88 | 0.88 | 0.88 |
|  |  | *sd (w)* | *0.26* | *0.26* | *0.25* | *0.25* | *0.25* |
| **Psychological demands** | **low-LMP** | mean (w) | 1.40 | 1.41 | 1.44 | 1.42 | 1.47 |
|  |  | *sd (w)* | *0.32* | *0.31* | *0.32* | *0.31* | *0.31* |
|  | **middle-LMP** | mean (w) | 1.41 | 1.43 | 1.46 | 1.48 | 1.43 |
|  |  | *sd (w)* | *0.32* | *0.31* | *0.31* | *0.30* | *0.30* |
|  | **high-LMP** | mean (w) | 1.44 | 1.46 | 1.47 | 1.45 | 1.46 |
|  |  | *sd (w)* | *0.31* | *0.29* | *0.29* | *0.28* | *0.29* |
| **Skill discretion** | **low-LMP** | mean (w) | 1.58 | 1.55 | 1.56 | 1.55 | 1.58 |
|  |  | *sd (w)* | *0.28* | *0.29* | *0.29* | *0.29* | *0.28* |
|  | **middle-LMP** | mean (w) | 1.68 | 1.63 | 1.65 | 1.61 | 1.66 |
|  |  | *sd (w)* | *0.28* | *0.29* | *0.29* | *0.30* | *0.29* |
|  | **high-LMP** | mean (w) | 1.68 | 1.68 | 1.71 | 1.68 | 1.70 |
|  |  | *sd (w)* | *0.28* | *0.29* | *0.27* | *0.28* | *0.28* |
| **Decision authority** | **low-LMP** | mean (w) | 1.67 | 1.62 | 1.60 | 1.64 | 1.65 |
|  |  | *sd (w)* | *0.39* | *0.41* | *0.41* | *0.41* | *0.40* |
|  | **middle-LMP** | mean (w) | 1.65 | 1.65 | 1.63 | 1.62 | 1.68 |
|  |  | *sd (w)* | *0.40* | *0.39* | *0.39* | *0.40* | *0.38* |
|  | **high-LMP** | mean (w) | 1.75 | 1.74 | 1.74 | 1.72 | 1.72 |
|  |  | *sd (w)* | *0.35* | *0.35* | *0.35* | *0.35* | *0.35* |

*Notes. Own calculations based on EWCS. Mean and standard deviation are weighted. Weights provided by EWCS. Sample size 74,959. Psychological demands, skill discretion and decision authority are standardized and lie between 1 and 2; job strain lies between 0.5 and 2. Higher values correspond to higher psych. demand/skill disc/dec auth/job strain.*

**Table A3. Predicted values of psychological demands by occupation and country group.**

|  | **1995** | **2000** | **2005** | **2010** | **2015** | **AME 2005 vs. 1995 (p-value)** | **AME 2015 vs. 2005 (p-value)** |
| --- | --- | --- | --- | --- | --- | --- | --- |
|  |  |  |  |  |  |  |  |
| **Psychological demands** | | | | | | | |
| **low-LMP** | | | | | | | |
| **High-skilled clerical** | 1.384 | 1.407 | 1.429 | 1.417 | 1.467 | 0.045 | 0.039 |
|  | *(1.324 - 1.444)* | *(1.348 - 1.467)* | *(1.374 - 1.483)* | *(1.364 - 1.470)* | *(1.414 - 1.521)* | *(0.087)* | *(0.105)* |
| **Low-skilled clerical** | 1.377 | 1.357 | 1.443 | 1.378 | 1.449 | 0.065 | 0.006 |
|  | *(1.318 - 1.437)* | *(1.299 - 1.415)* | *(1.388 - 1.498)* | *(1.326 - 1.431)* | *(1.396 - 1.503)* | *(0.012)* | *(0.786)* |
| **High-skilled manual** | 1.380 | 1.411 | 1.499 | 1.449 | 1.516 | 0.119 | 0.017 |
|  | *(1.319 - 1.441)* | *(1.352 - 1.471)* | *(1.441 - 1.558)* | *(1.393 - 1.505)* | *(1.460 - 1.573)* | *(0.000)* | *(0.534)* |
| **Low-skilled manual** | 1.361 | 1.407 | 1.426 | 1.448 | 1.496 | 0.065 | 0.071 |
|  | *(1.301 - 1.421)* | *(1.347 - 1.466)* | *(1.371 - 1.480)* | *(1.394 - 1.503)* | *(1.442 - 1.551)* | *(0.015)* | *(0.004)* |
| **AME LM vs. HC (p-value)** | -0.023 | -0.001 | -0.003 | 0.031 | 0.029 |  |  |
|  | *(0.108)* | *(0.957)* | *(0.818)* | *(0.016)* | *(0.008)* |  |  |
| **middle-LMP** | | | | | | | |
| **High-skilled clerical** | 1.385 | 1.421 | 1.439 | 1.442 | 1.416 | 0.055 | -0.023 |
|  | *(1.331 - 1.438)* | *(1.368 - 1.474)* | *(1.384 - 1.495)* | *(1.382 - 1.502)* | *(1.356 - 1.476)* | *(0.041)* | *(0.337)* |
| **Low-skilled clerical** | 1.375 | 1.389 | 1.412 | 1.433 | 1.423 | 0.037 | 0.011 |
|  | *(1.322 - 1.428)* | *(1.335 - 1.442)* | *(1.356 - 1.468)* | *(1.374 - 1.493)* | *(1.363 - 1.483)* | *(0.166)* | *(0.649)* |
| **High-skilled manual** | 1.456 | 1.474 | 1.474 | 1.525 | 1.462 | 0.018 | -0.012 |
|  | *(1.401 - 1.511)* | *(1.420 - 1.529)* | *(1.415 - 1.533)* | *(1.463 - 1.587)* | *(1.399 - 1.525)* | *(0.532)* | *(0.660)* |
| **Low-skilled manual** | 1.395 | 1.465 | 1.464 | 1.507 | 1.452 | 0.070 | -0.013 |
|  | *(1.340 - 1.449)* | *(1.411 - 1.519)* | *(1.407 - 1.522)* | *(1.446 - 1.568)* | *(1.390 - 1.513)* | *(0.014)* | *(0.626)* |
| **AME LM vs. HC (p-value)** | 0.010 | 0.044 | 0.025 | 0.065 | 0.036 |  |  |
|  | *(0.448)* | *(0.000)* | *(0.073)* | *(0.000)* | *(0.002)* |  |  |
| **high-LMP** | | | | | | | |
| **High-skilled clerical** | 1.444 | 1.447 | 1.497 | 1.459 | 1.481 | 0.052 | -0.016 |
|  | *(1.391 - 1.497)* | *(1.394 - 1.501)* | *(1.444 - 1.550)* | *(1.404 - 1.515)* | *(1.427 - 1.535)* | *(0.033)* | *(0.491)* |
| **Low-skilled clerical** | 1.418 | 1.431 | 1.468 | 1.436 | 1.459 | 0.050 | -0.009 |
|  | *(1.365 - 1.471)* | *(1.378 - 1.485)* | *(1.414 - 1.521)* | *(1.379 - 1.492)* | *(1.404 - 1.514)* | *(0.047)* | *(0.717)* |
| **High-skilled manual** | 1.451 | 1.475 | 1.523 | 1.438 | 1.471 | 0.072 | -0.052 |
|  | *(1.395 - 1.507)* | *(1.419 - 1.531)* | *(1.465 - 1.581)* | *(1.379 - 1.498)* | *(1.412 - 1.531)* | *(0.013)* | *(0.074)* |
| **Low-skilled manual** | 1.417 | 1.443 | 1.466 | 1.458 | 1.439 | 0.049 | -0.027 |
|  | *(1.362 - 1.471)* | *(1.388 - 1.498)* | *(1.409 - 1.522)* | *(1.401 - 1.516)* | *(1.383 - 1.496)* | *(0.077)* | *(0.320)* |
| **AME LM vs. HC (p-value)** | -0.028 | -0.004 | -0.031 | -0.001 | -0.042 |  |  |
|  | *(0.033)* | *(0.699)* | *(0.034)* | *(0.916)* | *(0.000)* |  |  |
| **Observations** | 74,959 | | | | | | |

*Notes: Predicted values based on multilevel model regressions with three levels (level 1: individual, level 2: country-years, level 3: country). Covariates included in the regression: gender, age (<30, 30-50, 50<), contract type (indefinite, fixed term, temporary, apprenticeship, other), nace (5 groups: "agriculture, hunting, forestry and fishing", "industry", "services", "public administration and defence; compulsory social sec", "other services"), GDP, three-way interactions of wave dummies, occupation (4 groups) and country groups (3 groups). 95% confidence intervals in parenthesis. AME: p-values in parenthesis. Sample: EU15, Waves included: 1995, 2000, 2005, 2010, 2015.*

**Table A4: Predicted values of skill discretion by occupation and country group.**

|  | **1995** | **2000** | **2005** | **2010** | **2015** | **AME 2005 vs. 1995 (p-value)** | **AME 2015 vs. 2005 (p-value)** |
| --- | --- | --- | --- | --- | --- | --- | --- |
|  |  |  |  |  |  |  |  |
| **Skill discretion** | | | | | | | |
| **low-LMP** | | | | | | | |
| **High-skilled clerical** | 1.680 | 1.667 | 1.694 | 1.704 | 1.696 | 0.013 | 0.003 |
|  | *(1.640 - 1.721)* | *(1.628 - 1.706)* | *(1.657 - 1.730)* | *(1.668 - 1.739)* | *(1.661 - 1.731)* | *(0.476)* | *(0.876)* |
| **Low-skilled clerical** | 1.576 | 1.516 | 1.554 | 1.516 | 1.524 | -0.022 | -0.030 |
|  | *(1.536 - 1.616)* | *(1.478 - 1.554)* | *(1.518 - 1.591)* | *(1.481 - 1.551)* | *(1.489 - 1.559)* | *(0.242)* | *(0.075)* |
| **High-skilled manual** | 1.492 | 1.481 | 1.488 | 1.477 | 1.530 | -0.005 | 0.042 |
|  | *(1.451 - 1.533)* | *(1.441 - 1.521)* | *(1.447 - 1.528)* | *(1.439 - 1.516)* | *(1.491 - 1.568)* | *(0.831)* | *(0.043)* |
| **Low-skilled manual** | 1.419 | 1.351 | 1.444 | 1.382 | 1.394 | 0.024 | -0.049 |
|  | *(1.379 - 1.460)* | *(1.312 - 1.391)* | *(1.407 - 1.480)* | *(1.346 - 1.419)* | *(1.358 - 1.430)* | *(0.204)* | *(0.005)* |
| **AME LM vs. HC (p-value)** | -0.261 | -0.316 | -0.250 | -0.321 | -0.302 |  |  |
|  | *(0.000)* | *(0.000)* | *(0.000)* | *(0.000)* | *(0.000)* |  |  |
| **middle-LMP** | | | | | | | |
| **High-skilled clerical** | 1.754 | 1.728 | 1.753 | 1.730 | 1.763 | -0.001 | 0.010 |
|  | *(1.719 - 1.790)* | *(1.693 - 1.763)* | *(1.716 - 1.790)* | *(1.691 - 1.769)* | *(1.724 - 1.803)* | *(0.953)* | *(0.552)* |
| **Low-skilled clerical** | 1.648 | 1.588 | 1.619 | 1.563 | 1.609 | -0.030 | -0.010 |
|  | *(1.613 - 1.684)* | *(1.553 - 1.623)* | *(1.582 - 1.656)* | *(1.523 - 1.602)* | *(1.570 - 1.649)* | *(0.114)* | *(0.577)* |
| **High-skilled manual** | 1.584 | 1.565 | 1.594 | 1.561 | 1.628 | 0.010 | 0.034 |
|  | *(1.547 - 1.621)* | *(1.528 - 1.602)* | *(1.554 - 1.635)* | *(1.519 - 1.602)* | *(1.585 - 1.671)* | *(0.624)* | *(0.102)* |
| **Low-skilled manual** | 1.495 | 1.433 | 1.458 | 1.407 | 1.450 | -0.037 | -0.009 |
|  | *(1.459 - 1.532)* | *(1.396 - 1.469)* | *(1.419 - 1.498)* | *(1.366 - 1.447)* | *(1.409 - 1.490)* | *(0.071)* | *(0.649)* |
| **AME LM vs. HC (p-value)** | -0.259 | -0.295 | -0.295 | -0.323 | -0.314 |  |  |
|  | *(0.000)* | *(0.000)* | *(0.000)* | *(0.000)* | *(0.000)* |  |  |
| **high-LMP** | | | | | | | |
| **High-skilled clerical** | 1.787 | 1.790 | 1.800 | 1.792 | 1.811 | 0.013 | 0.011 |
|  | *(1.752 - 1.822)* | *(1.756 - 1.825)* | *(1.765 - 1.835)* | *(1.756 - 1.829)* | *(1.776 - 1.847)* | *(0.452)* | *(0.476)* |
| **Low-skilled clerical** | 1.671 | 1.640 | 1.695 | 1.626 | 1.644 | 0.024 | -0.051 |
|  | *(1.635 - 1.706)* | *(1.605 - 1.675)* | *(1.660 - 1.730)* | *(1.589 - 1.664)* | *(1.608 - 1.680)* | *(0.171)* | *(0.003)* |
| **High-skilled manual** | 1.625 | 1.639 | 1.642 | 1.619 | 1.655 | 0.017 | 0.014 |
|  | *(1.586 - 1.663)* | *(1.601 - 1.676)* | *(1.601 - 1.682)* | *(1.579 - 1.660)* | *(1.614 - 1.697)* | *(0.438)* | *(0.525)* |
| **Low-skilled manual** | 1.487 | 1.480 | 1.485 | 1.507 | 1.492 | -0.002 | 0.007 |
|  | *(1.450 - 1.524)* | *(1.443 - 1.516)* | *(1.446 - 1.524)* | *(1.469 - 1.546)* | *(1.454 - 1.530)* | *(0.915)* | *(0.718)* |
| **AME LM vs. HC (p-value)** | -0.300 | -0.311 | -0.315 | -0.285 | -0.320 |  |  |
|  | *(0.000)* | *(0.000)* | *(0.000)* | *(0.000)* | *(0.000)* |  |  |
| **Observations** | 74,959 | | | | | | |

*Notes: Predicted values based on multilevel model regressions with three levels (level 1: individual, level 2: country-years, level 3: country). Covariates included in the regression: gender, age (<30, 30-50, 50<), contract type (indefinite, fixed term, temporary, apprenticeship, other), nace (5 groups: "agriculture, hunting, forestry and fishing", "industry", "services", "public administration and defence; compulsory social sec", "other services"), GDP, three-way interactions of wave dummies, occupation (4 groups) and country groups (3 groups). 95% confidence intervals in parenthesis. AME: p-values in parenthesis. Sample: EU15, Waves included: 1995, 2000, 2005, 2010, 2015.*

**Table A5: Predicted values of decision authority by occupation and country group.**

|  | **1995** | **2000** | **2005** | **2010** | **2015** | **AME 2005 vs. 1995 (p-value)** | **AME 2015 vs. 2005 (p-value)** |
| --- | --- | --- | --- | --- | --- | --- | --- |
|  |  |  |  |  |  |  |  |
| **Decision authority** | | | | | | | |
| **low-LMP** | | | | | | | |
| **High-skilled clerical** | 1.755 | 1.736 | 1.728 | 1.764 | 1.700 | -0.028 | -0.028 |
|  | *(1.693 - 1.818)* | *(1.675 - 1.797)* | *(1.671 - 1.785)* | *(1.708 - 1.820)* | *(1.645 - 1.756)* | *(0.289)* | *(0.235)* |
| **Low-skilled clerical** | 1.678 | 1.606 | 1.580 | 1.580 | 1.566 | -0.098 | -0.014 |
|  | *(1.616 - 1.740)* | *(1.546 - 1.666)* | *(1.523 - 1.638)* | *(1.525 - 1.635)* | *(1.511 - 1.621)* | *(0.000)* | *(0.539)* |
| **High-skilled manual** | 1.549 | 1.526 | 1.507 | 1.507 | 1.569 | -0.042 | 0.061 |
|  | *(1.486 - 1.613)* | *(1.464 - 1.589)* | *(1.444 - 1.570)* | *(1.447 - 1.567)* | *(1.509 - 1.628)* | *(0.157)* | *(0.033)* |
| **Low-skilled manual** | 1.571 | 1.432 | 1.506 | 1.500 | 1.488 | -0.064 | -0.018 |
|  | *(1.508 - 1.634)* | *(1.370 - 1.493)* | *(1.449 - 1.564)* | *(1.443 - 1.558)* | *(1.432 - 1.545)* | *(0.015)* | *(0.462)* |
| **AME LM vs. HC (p-value)** | -0.185 | -0.304 | -0.222 | -0.264 | -0.212 |  |  |
|  | *(0.000)* | *(0.000)* | *(0.000)* | *(0.000)* | *(0.000)* |  |  |
| **middle-LMP** | | | | | | | |
| **High-skilled clerical** | 1.795 | 1.770 | 1.758 | 1.739 | 1.773 | -0.038 | 0.015 |
|  | *(1.739 - 1.851)* | *(1.715 - 1.826)* | *(1.700 - 1.815)* | *(1.678 - 1.800)* | *(1.712 - 1.834)* | *(0.147)* | *(0.511)* |
| **Low-skilled clerical** | 1.687 | 1.634 | 1.648 | 1.613 | 1.656 | -0.039 | 0.008 |
|  | *(1.632 - 1.743)* | *(1.578 - 1.689)* | *(1.590 - 1.706)* | *(1.552 - 1.674)* | *(1.594 - 1.717)* | *(0.128)* | *(0.749)* |
| **High-skilled manual** | 1.624 | 1.620 | 1.617 | 1.596 | 1.653 | -0.006 | 0.036 |
|  | *(1.566 - 1.681)* | *(1.563 - 1.678)* | *(1.555 - 1.680)* | *(1.531 - 1.660)* | *(1.587 - 1.719)* | *(0.825)* | *(0.218)* |
| **Low-skilled manual** | 1.507 | 1.497 | 1.533 | 1.514 | 1.550 | 0.025 | 0.017 |
|  | *(1.450 - 1.565)* | *(1.440 - 1.555)* | *(1.472 - 1.594)* | *(1.452 - 1.577)* | *(1.486 - 1.613)* | *(0.379)* | *(0.519)* |
| **AME LM vs. HC (p-value)** | -0.288 | -0.273 | -0.225 | -0.225 | -0.223 |  |  |
|  | *(0.000)* | *(0.000)* | *(0.000)* | *(0.000)* | *(0.000)* |  |  |
| **high-LMP** | | | | | | | |
| **High-skilled clerical** | 1.814 | 1.818 | 1.780 | 1.785 | 1.800 | -0.035 | 0.020 |
|  | *(1.759 - 1.869)* | *(1.762 - 1.873)* | *(1.724 - 1.835)* | *(1.728 - 1.842)* | *(1.744 - 1.855)* | *(0.142)* | *(0.361)* |
| **Low-skilled clerical** | 1.753 | 1.718 | 1.721 | 1.664 | 1.669 | -0.032 | -0.052 |
|  | *(1.697 - 1.808)* | *(1.663 - 1.773)* | *(1.665 - 1.777)* | *(1.606 - 1.722)* | *(1.612 - 1.726)* | *(0.195)* | *(0.025)* |
| **High-skilled manual** | 1.728 | 1.714 | 1.682 | 1.632 | 1.702 | -0.046 | 0.020 |
|  | *(1.669 - 1.788)* | *(1.655 - 1.772)* | *(1.620 - 1.744)* | *(1.569 - 1.695)* | *(1.639 - 1.766)* | *(0.125)* | *(0.508)* |
| **Low-skilled manual** | 1.613 | 1.599 | 1.619 | 1.598 | 1.598 | 0.007 | -0.021 |
|  | *(1.555 - 1.671)* | *(1.542 - 1.657)* | *(1.559 - 1.680)* | *(1.538 - 1.658)* | *(1.539 - 1.658)* | *(0.818)* | *(0.443)* |
| **AME LM vs. HC (p-value)** | -0.202 | -0.218 | -0.160 | -0.187 | -0.201 |  |  |
|  | *(0.000)* | *(0.000)* | *(0.000)* | *(0.000)* | *(0.000)* |  |  |
| **Observations** | 74,959 | | | | | | |

*Notes: Predicted values based on multilevel model regressions with three levels (level 1: individual, level 2: country-years, level 3: country). Covariates included in the regression: gender, age (<30, 30-50, 50<), contract type (indefinite, fixed term, temporary, apprenticeship, other), nace (5 groups: "agriculture, hunting, forestry and fishing", "industry", "services", "public administration and defence; compulsory social sec", "other services"), GDP, three-way interactions of wave dummies, occupation (4 groups) and country groups (3 groups). 95% confidence intervals in parenthesis. AME: p-values in parenthesis. Sample: EU15, Waves included: 1995, 2000, 2005, 2010, 2015.*
